# Authors' reply

**DOI:** 10.1192/bjb.2021.78

**Published:** 2021-10

**Authors:** Lucy Griffin, Katie Clyde, Richard Byng, Susan Bewley

**Affiliations:** Psychiatrist, Royal College of Psychiatrists, UK. Email: drlucygriffin@outlook.com

We thank Dr MacFarlane for his response^1^ and welcome his comments about various providers and pharmaceutical agencies as well as freedom of speech.

We did not review the neuroscience of brain sex differences but draw interested readers’ attention to a recent review^2^ and accessible analyses of available research.^3,4^ We agree that the art of psychiatric diagnosis depends on the clinician accepting the truth of the patient's own experience. In the absence of objective diagnostic tests, believing and trusting the patient's own subjective narrative is central to the doctor–patient relationship. However, this starts to lose coherence when the doctor must readjust their own understanding of material reality in order to accommodate another's subjective belief. Declaring that ‘Despite having the body of a man, I am in all other respects a woman’ supposes some inherent essence of gender that many would reject. Reorganising psychiatry to give primacy of gender identity over sex risks breaching the necessary boundaries that exist to maintain the safety and dignity of individuals, groups of people and society more generally.

Without a clear definition of conversion therapy, it is not possible to know the extent of the practice in the UK. Proponents of affirmative care have argued that conversion therapy is anything that might act as a barrier to medical transition.^5,6^ It would follow that attempts to assess and treat coexistent mental illness, or even the process of making an accurate initial diagnosis of gender dysphoria, could be described as conversion therapy rather than the basic standard of clinical care that would apply for any other presentation.^7^

The authors remain opposed to any treatment model designed to coercively alter the sexual orientation of bisexuals, gay men or lesbians. It is crucial to distinguish between sexual orientation and gender identity when the latter comes with an expectation for complex, irreversible medical interventions, described as ‘affirmative’. If sustained, long-term benefits of medical and surgical transition could be clearly and independently demonstrated, it would be appropriate to offer these interventions early, but the evidence is not convincing.^8^ Therefore it is reasonable to exercise therapeutic caution, especially in light of growing concerns about complications and regret,^9^ particularly in younger patients.

Given government moves to criminalise conversion therapy in medical settings, the nature of ‘barriers to treatment’ must be clearly described.^9^ New laws will need detailed supplementary guidance for the benefit of patients, doctors and the criminal justice system. We propose that organisations representing clinicians should help legislators make explicit that neutrally framed therapeutic or exploratory work is not conversion therapy, irrespective of how an individual ultimately feels about their own identity.

In the absence of evidence-based guidelines underpinned by solid research, we cannot make recommendations about treatment pathways, and do not advocate one particular model over any other. We draw readers’ attention to the unexplained increase in referral numbers, the higher numbers of children and young people seeking interventions, and the shift in sex ratio,^10^ as such demographic changes are significant and deserving of research and explanation. Doctors should ‘first do no harm’. The bar for informed consent to life-changing, irreversible medical and surgical interventions is necessarily high.^9^ Enhanced service provision and new care pathways should be informed by robust research in this patient group.

## References

[1] Griffin L, Clyde K, Byng R, Bewley S. Sex, gender and gender identity: a re-evaluation of the evidence. BJPsych Bull 2020. Available from: 10.1192/bjb.2020.73. PMC8596152 32690121

[2] McCarthy MM. Multifaceted origins of sex differences in the brain. Philos Trans R Soc B Biol Sci 2016; 371(1688): 20150106. 10.1098/rstb.2015.0106 PMC4785894 26833829

[3] Fine C. Delusions of Gender: The Real Science Behind Sex Differences. Icon Books, 2011.

[4] Rippon G. The Gendered Brain: The New Neuroscience that Shatters the Myth of the Female Brain. Vintage, 2020.

[5] Royal College of Psychiatrists. Supporting Transgender and Gender-Diverse People. Royal College of Psychiatrists, 2018. Available from: https://www.rcpsych.ac.uk/docs/default-source/improving-care/better-mh-policy/position-statements/ps02_18.pdf?sfvrsn=af4d4aad_4.

[6] Edinburgh Action for Trans Health. *Trans Health Manifesto*. 2017. Available from: https://edinburghath.tumblr.com/post/163521055802/trans-health-manifesto [cited 18 Mar 2021].

[7] General Medical Council. Good Medical Practice. General Medical Council, 2013.

[8] Griffin L, Clyde K, Byng R, Bewley S. *Analysis of Cited Publications*. 2021. Available from: https://figshare.com/articles/figure/Analysis_of_cited_publications_pdf/13626380 [cited 15 Feb 2021].

[9] *Bell v Tavistock* [2020] EWHC 3274. Available from: https://www.judiciary.uk/wp-content/uploads/2020/12/Bell-v-Tavistock-Clinic-and-ors-Summary.pdf.

[10] de Graaf NM, Giovanardi G, Zitz C, Carmichael P. Sex ratio in children and adolescents referred to the gender identity development service in the UK (2009–2016). Arch Sex Behav 2018; 47(5): 1301–4. 29696550 10.1007/s10508-018-1204-9

